# Management of tibial shaft fractures with closed intra medullary interlocking nail among Indians

**DOI:** 10.6026/97320630018562

**Published:** 2022-06-30

**Authors:** Kadiri Venkata Ranganath, Vinyas Mayasa, Vijay Narasimman Reddy, Manoj Prathap Chandran, N Anuradha, S Karthikeyan

**Affiliations:** 1 Department of Orthopaedics, SriDevaraj Urs Medical College and Hospital, Kolar 563101, Karnataka, India; 2 Department of Pharmacology, MNR College of Pharmacy, Sangareddy, Telangana, India; 3 Department of Orthopaedics, SreeBalaji Medical College and Hospital, Chromepet, Chennai - 600044, India; 4 Department of Orthodontics, SreeBalaji Dental College and Hospital, Pallikaranai, Chennai - 600100, India; 5 Department of General Medicine, Sree Balaji Medical College and Hospital, Chromepet, Chennai - 600044, India; 6 Department of General Surgery, SreeBalaji Medical College and Hospital, Chromepet, Chennai - 600044, India

**Keywords:** Isolated tibial shaft fracture, fibula intact, intramedullary interlocking nail, union, delayed union

## Abstract

It is of interest to document data on the well Reamed Intramedullary Nailing in Isolated Tibial Diaphyseal Fractures without Fibular Osteotomy among Indians. 120 patients with isolated tibial diaphyseal fractures were treated with IMIL nail (84 closed fractures, 16 type I open fractures, and 20 type II open fractures) were involved in this study. Research was carried out over a five-and-a-half-year period, from July 2013 to December 2018.According to Johner and Wruh's criteria, good functional findings were achieved in 70% of patients, better operational results in 15%, reasonable functional results in 5%, and poor functional results in 10% of cases after surgery. The percentage of union in the present analysis was 90%. The average time for union was 5 months, with 84 fractures healing before 5 months. Intramedullary Interlocking Nailing reduces length of stay in hospital, lowers the financial load, and promotes early return to work without the need for further surgical treatment such as partial fibular osteotomy.

## Background:

Most orthopaedic trauma surgeons consider tibial shaft fractures to be among the most common major bone injuries. Tibial fractures with a healthy fibula having attracted the interest of orthopaedic trauma surgeons everywhere around the world for a long time. Not only are they frequent, but they're also notoriously tough to treat. When compared to other long bone injuries, the subcutaneous placement of the tibia on the anteromedial surface means that severe bone and soft tissue injuries are common, and there is a very high frequency of open fractures [1]. Tibio-fibular length disparity develops when the fibula bone remains intact, causing different strain patterns in both the tibia and fibula. Such changes can result in tibial bone delayed union, malunion, or non union, and also knee and ankle joint issues. It's probable that the reduced incidence of problems in people under the age of twenty is because of the increased flexibility of the fibulae and soft tissues around their joints [2]. Decrease of the tibia as well as an abnormally high rate of varusmal union and non union is challenges in Orthopaedic treatment of leg fractures with fibula intact. The most reliable approach for treating tibial shaft fractures with an intact fibula is intramedullary nailing [3]. Tibial diaphysis fractures with the fibula intact have always piqued the interest of orthopaedic surgeons all over the world. The question of whether tibial diaphyseal fractures with intact fibula have been connected with a good or worse diagnosis has indeed been debated for a long time [4-5]. The prime reason of deferred union or non union was dislocation of even more than 50percent of the thickness of the tibial bone at the injured area. In fractures with more over 50percent initial velocity, decrease has been hard to maintain, and fracture comminution delayed fracture healing in many cases. Fractures that have more than 50% comminution are called unstable and are often linked to high-energy trauma. Misplaced Tibial diaphyseal fractures with intact fibula are treated by closed intramedullary interlocking. Nailing with reaming produces better functional effects than utilising a cast [6]. Various investigations have found that A minimally displaced Tibial shaft fracture with an undamaged fibula has a very good prognosis [7-9]. According to Charnley, the fibula bone doesn't break because it's naturally flexible and the ligaments that hold it in place are very flexible. Even though the initial force may be enough to break the Tibia bone and tear the local soft tissues, the fibula bone doesn't break because of these factors. He also mentioned that radiographs frequently fail to indicate the real degree of the displacement incurred at the time of trauma, and hence this type of fracture pattern can be prone to numerous difficulties [10,11]. Complications such as delayed and non-union are common in tibial diaphyseal fractures with an intact fibula [12] are known. Tibial diaphyseal fractures will continue to be treated with intramedullary interlocking nailing. However, new plates are becoming less intrusive as they are associated with increased soft tissue stripping and probable devascularisation [13]. Therefore, it is of interest to document data on the management of tibial shaft fractures with closed intra medullary interlocking nail among Indians.

## Materials and methods:

After getting institutional ethical clearance and the subjects' permission, our prospective analysis was carried on 120 patients in a tertiary trauma care centre over a five-and-a-half-year period with regular follow-up. The study enrolled 120 people with Tibial Diaphyseal Injuries Between July 2013 and December 2018 who had underwent surgery (IMIL nail) at Sri Devaraj Urs Medical College & Hospital in Kolar, state of Karnataka.

## Inclusion criteria:

[1] Closed isolated Tibial shaft fractures.

[2] Type I, Type II, and Type IIIA open tibial fractures with intact fibulas as defined by Gustillo-Anderson grading.

[3] Tibial injuries with an intact fibula in people above the age of 18.

## Criteria for Exclusion:

[1] Intra-articular fractures of Tibia

[2] Pathological fractures of Tibia.

At the end of the six-month post-operative period, Johner and Wruh's criteria were used to conduct a follow-up and assessment. The tibial tubercle–medial malleolar distance is used to calculate the nail length prior to surgery (TMD). The patient began active knee and ankle mobilisation exercises on the first post-operative day after the surgery. Patients were allowed to walk with crutches on the second post-operative day only if they had concurrent injuries and if their overall health and tolerance allowed it.On the 14th postoperative day, the skin sutures were removed. Partially weight bearing with crutch walking was started later, based on the type of fractures, stiffness of fixation, and associated ailments. Patients were examined clinically and radio graphically according to the conventional Performa protocol at intervals of 6 weeks, 12 weeks, 18 weeks, and 24 weeks.

## Statistics:

Statistical analysis was done with the use of parametric student T test and chi square test. Statistically significant was defined as P=0.0001

## Results:

Patients were followed for 21.90 months on average (range 10-54), which is statistically significant (p = 0.0001). At the conclusion of six months, a detailed study of functional results was performed on the patients using Johner and Wruh's criteria. The statistical significance of the p value is 0.0001.84 out of 120 patients had outstanding outcomes, corresponding to 70%, which is statistically significant (p = 0.0001), 18 patients had good results, corresponding to 15%, 6 patients had fair results, corresponding to 5%, and 12 patients had poor results, corresponding to 10%. Our patients were mostly between the ages of 18 and 40, with 73.3 percent being within those ages. Our study's youngest patient was 19 years old, and the oldest patient was 60 years old. Only ten individuals in the study had other injuries with five having metatarsals and phalanges fractures mended with k wires, another having a chest injury treated conservatively, and the remaining four having head injuries. The remaining 110 patients had no associated injuries. In our investigation, 108 out of 120 isolated tibial shaft fractures had united satisfactorily, resulting in a percentage of union rate of around 90%, which was statistically significant (p=0.0001), indicating a better outcome. Despite the fact that most tibial shaft fractures are supposed to cure within 24 weeks, in our study, 55.8% (67 instances) of isolated tibial shaft fractures healed well within 5 months, or approximately 20 weeks which was statistically significant (p=0.0001)

## Discussion:

The focus of this research is to see how single tibial shaft injuries repaired with intramedullary nailing rather than fibula osteotomy fare in the long run. It can assist surgeons in providing more accurate information to patients on the long-term function of a tibia fracture with just an intact fibula after intramedullary nailing. In the face of various treatment options such as external fixation, functional cast bracing, and internal plate and screw fixation, it also emphasizes the need of intramedullary interlocking nailing for the treatment of isolated tibial shaft fractures. A total of 120 patients with isolated tibial shaft fractures were treated with closed intramedullary interlocking nailing in the current investigation. For a period of six months, they were all followed up on. The patients in the study were of various ages, were of both sexes, and the fractures were of various sorts and levels. The intramedullary interlocking nail improves length, alignment, rotation control, periosteal blood supply, some endosteal blood supply, biological osteo synthesis, and infection and malunion rates. The locking screws have the advantage over other traditional procedures in that they limit the rate of malunion, prevent fracture alignment loss, angulation, and shortening, all of which are frequent problems with functional braces and plaster casts. All of the patients in the following study were 35 years old on average. Isolated tibial shaft fractures were more prevalent in people between the ages of 18 and 39. Active young people were the hardest hit. The majority are working adult men who participate in outdoor sports. The most common cause of tibial shaft fractures was a traffic accident. In 87 of the 120 patients, mid diaphyseal tibial injuries has been the most frequent kind of tibial fracture, accounting for 73.3 percent of the total. The duration of union in our study ranged from 4 to 9 months, with an average of 5 months. Even though most tibial shaft injuries are expected to heal in 24 weeks, in our analysis, 67 of them recovered well before 5 months, around 20 weeks, which was statistically significant (p=0.0001) and indicated a better prognosis. Twenty-seven patients out of a total of 120 were admitted for delayed union, accounting for around a quarter of the cases. Closed isolated tibial fractures heal quickly with IMIL nailing without fibular osteotomy. Dynamization and bone marrow injections were used to successfully handle all of the delayed union cases. In our studies, displaced and comminuted fractures appear to become a source of prolonged union and nonunion. Isolated tibial diaphyseal fractures with intact fibula, especially in patients over the age of 20, are frequently associated with delayed or non-union of the fracture, according to the findings [Figure 1,2 - see PDF] In the following study, 27 out of 120 patients (or 23 percent of all patients) opted for delayed union. Dynamization and bone marrow injections at the fracture site were used to treat all of the fractures that had a prolonged union. A limited range of motion of the ankle was found in 40% of the cases (48 instances). Persistent quadriceps atrophy was seen in 32 patients (27 percent), and calf atrophy was seen at the same rate. There was no discomfort to anterior knee palpation in 95 subjects (75.8%). Despite the absence of radiographic malalignment, 42 (35.4%) of the 31 patients radio graphically assessed revealed signs of arthritis.

## Conclusion:

Surgical fixation of Tibial fractures with IMIL nailing alone with no need for fibular osteotomy heals quickly & well in isolated tibial fractures. Isolated tibial diaphyseal fractures for closed & open type 1 injuries, the Intramedullary Interlocking Nail without Fibular Osteotomy is the most effective treatment option and united well before 24 weeks, while more displaced fractures have gone for delayed union. In displaced fractures, open type 2 fractures with intact fibula, delayed union is common, non union seen in open type 3 fractures . Data shows that long-term physical disability may not be a problematic for isolated tibial shaft fracture patients and it should be considered when providing prognostic information to pts.
